# Free Thyroxine During Early Pregnancy and Risk for Gestational Diabetes

**DOI:** 10.1371/journal.pone.0149065

**Published:** 2016-02-24

**Authors:** James E. Haddow, Wendy Y. Craig, Louis M. Neveux, Glenn E. Palomaki, Geralyn Lambert-Messerlian, Fergal D. Malone, Mary E. D’Alton, First and Second Trimester Risk of Aneuploidy (FaSTER) Research Consortium

**Affiliations:** 1 Department of Pathology and Laboratory Medicine, Division of Medical Screening and Special Testing, Women and Infants Hospital and Alpert Medical School of Brown University, Providence, Rhode Island, United States of America; 2 Savjani Institute for Health Research, Windham, Maine, United States of America; 3 Maine Medical Center Research Institute, Scarborough, Maine, United States of America; 4 Department of Obstetrics & Gynecology, Division of Maternal-Fetal Medicine, University College of Physicians and Surgeons, New York, New York, United States of America; 5 Department of Obstetrics & Gynecology, Royal College of Surgeons in Ireland, Dublin, Ireland, United Kingdom; University of Barcelona, SPAIN

## Abstract

Several studies have now reported associations between gestational diabetes mellitus (GDM) and low free thyroxine (fT4) during the second and third trimesters, but not in the first trimester. The present study further examines relationships between low fT4, maternal weight, and GDM among women in the FaSTER (First and Second Trimester Evaluation of Risk) trial, in an effort to determine the extent to which thyroid hormones might contribute to causality. The FaSTER cohort includes 9351 singleton, euthyroid women; 272 of these women were subsequently classified as having GDM. Thyrotropin (TSH), fT4, and thyroid antibodies were measured at 11–14 weeks’ gestation (first trimester) and 15–18.9 weeks’ gestation (second trimester). An earlier report of this cohort documented an inverse relationship between fT4 in the second trimester and maternal weight. In the current analysis, women with GDM were significantly older (32 vs. 28 years) and weighed more (75 vs. 64.5 kg). Maternal weight and age (but not TSH) were significantly associated univariately with fT4 (dependent variable), in the order listed. Second trimester fT4 odds ratios (OR) for GDM were 2.06 [95% CI 1.37–3.09] (unadjusted); and 1.89 [95% CI 1.26–2.84] (adjusted). First trimester odds ratios were not significant: OR 1.45 [95%CI 0.97–2.16] (unadjusted) and 1.11 [95% CI 0.74–1.62] (adjusted). The second trimester fT4/GDM relationship thus appeared to strengthen as gestation progressed. In FaSTER, high maternal weight was associated with both low fT4 and a higher GDM rate in the second trimester. Peripheral deiodinase activity is known to increase with high caloric intake (represented by high weight). We speculate that weight-related low fT4 (the metabolically inactive prohormone) is a marker for deiodinase activity, serving as a substrate for conversion of fT4 to free triiodothyronine (fT3), the active hormone responsible for glucose-related metabolic activity.

## Introduction

In the United States, approximately 4% of pregnancies are diagnosed as having gestational diabetes mellitus (GDM) [1]. GDM resolves after delivery but recurs 30–50% of the time with subsequent pregnancies; longer term, type 2 diabetes occurs in up to 70% of women with a previous history of GDM [2, 3]. The pattern of post-delivery resolution of GDM, followed by recurrence with a subsequent pregnancy, suggests pregnancy-related stress. In spite of well-known associations with glucose intolerance, β-cell dysfunction, and insulin resistance, the pathogenesis of GDM is incompletely understood [4, 5]. Recently, several studies among euthyroid women have reported associations between GDM and low free thyroxine (fT4) during the second and third trimesters [6–9] but not in the first trimester [7, 9, 10] offering a clue that thyroid hormones may provide further insight into pathogenesis. Guzman-Gutiérrez et al. have recently proposed that the low level of fT4 associated with GDM may be compensated by increased placental availability of T3/T4 via elevation in the activity of thyroid hormone transporters and/or reduction in deiodinases in the feto-placental circulation [11].

The present study examines in greater depth the relationship between free thyroxine (fT4) concentration and gestational diabetes mellitus (GDM) among euthyroid women with a singleton pregnancy who participated in the First and Second Trimester Evaluation of Risk (FaSTER) trial [12]. In the initial analysis of that cohort in 2008, Cleary-Goldman et al explored whether relationships might exist between hypothyroxinemia (fT4 concentrations below the 2.5^th^ percentile) and several pregnancy/delivery complications [7]. Among euthyroid women, they found an association between hypothyroxinemia and subsequent GDM at 15–18.9 weeks’ gestation, but not at 11–14 weeks’ gestation. A later analysis of that same dataset focused on the impact of high fT4 concentrations (highest quintile) on birthweight at 15–18.9 weeks’ gestation and noted incidentally that the frequency of gestational diabetes ranged from 5% in the lowest fT4 quintile to 1.3% in the highest fT4 quintile [13].

Also relevant to the present study is the reciprocal relationship between fT4 and maternal weight documented in an earlier FaSTER report [14]. Weight and/or body mass index (BMI) are both established risk factors for GDM and need to be taken into account (along with other covariates) when examining the fT4/GDM relationship [15, 16]. The present analysis examines the fT4/GDM relationship identified in the FaSTER trial by exploring the extent to which fT4 might be an independent risk factor for GDM and speculating on how thyroid hormones might contribute to causality.

## Materials and Methods

The multicenter FaSTER trial, a National Institute of Child Health and Human Development-sponsored study, was a prospective multicenter investigation of singleton pregnancies from an unselected obstetric population at 15 centers throughout the United States, beginning in October 1, 1990 and ending in December 31, 2002 [7, 13, 14, 17]. Institutional review board approval for this study was granted by the Columbia University Medical Center, New York and by Women and Infants Hospital, Brown University, Rhode Island. After written informed consent was obtained, patients were enrolled between 10 3/7 and 13 6/7 weeks of gestation; weight and height were recorded on that occasion. All subjects had a live singleton intrauterine pregnancy without evidence of anencephaly or cystic hygroma, confirmed by ultrasonography at the time of trial enrollment.

Maternal serum samples were collected at two intervals during early pregnancy. At five recruitment centers, participants were asked to give supplementary consent to allow use of residual sample and pregnancy-related information for additional research. Women whose pregnancies were affected by Down syndrome or other chromosome structural abnormalities were excluded. Among women who consented, inclusion criteria for the present study required: 1) thyrotropin (TSH) and free T4 measurement available and gestational age established by ultrasound (10,074 women), 2) no known thyroid disease (9,670 women), 3) available weight and height (9,630 women), and 4) TSH measurements between 2^nd^ and 98^th^ centiles (9,351 women) at 11–14 weeks’ gestation (0.008–4.17 IU/L), and at 15–18.9 weeks’ gestation (0.05–3.80 IU/L).

Post-delivery follow-up was performed by the research coordinator at each site or by telephone interview. A single perinatologist and a pediatric geneticist reviewed maternal and pediatric medical records for the following patient subsets: abnormal first and/or second trimester screening, adverse obstetric or pediatric outcomes, and 10% of normal subjects randomly selected from the trial database. A purpose-designed computerized tracking system with up to 10 contacts per subject was used to ensure complete outcome collection. Pregnancy and pediatric outcomes were obtained in more than 98% of cases. Gestational diabetes was classified as two abnormal values on a 3-hour glucose challenge test.

Samples were stored at -80°C. Levels of TSH, free T4 and thyroperoxidase (TPO) antibodies were measured between July 2004 and May 2005 using the Immulite 2000 methodology (Siemens Medical Solutions Diagnostics, Tarrytown, NY). Normative data involving these analytes have been published separately [17]. The lower limit for interpreting TSH measurements was 0.01 mIU/L. Long-term coefficients of variation were 5.3%, 6.9% and 3.8% at TSH concentrations of 0.53, 4.5 and 21.9 mU/L; 8.1%, 6.5% and 7.9% at free T4 concentrations of 11.6, 23.2 and 41.2 pmol/L; 2.5%, 6.6% and 5.0% at TPO antibody concentrations of 30, 39 and 546 IU/mL.

### Statistical Analyses

**Fig 1:**
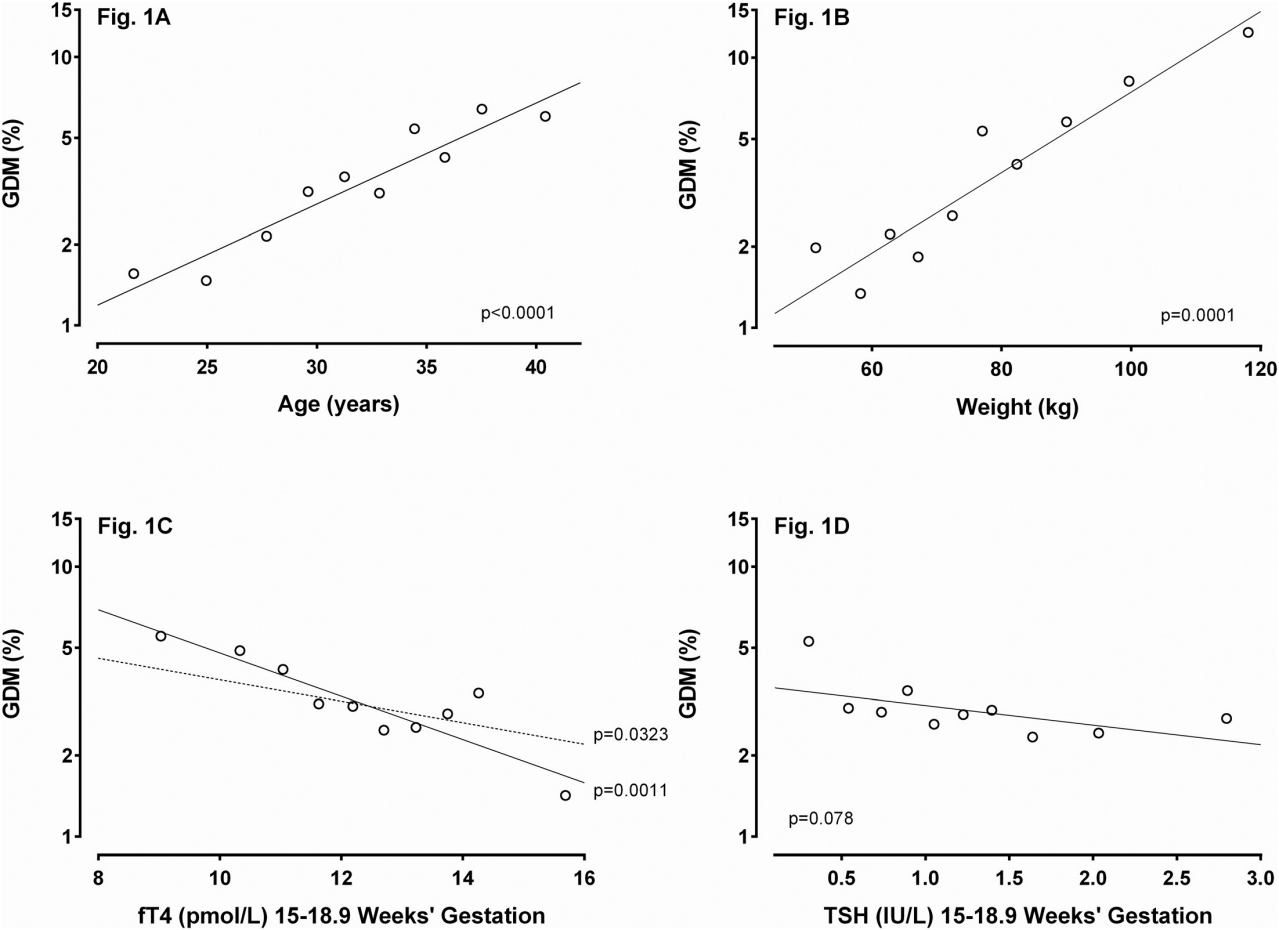
Relationships between gestational diabetes (GDM) and age (Fig 1A), weight (Fig 1B), free thyroxine (fT4) (Fig 1C), and thyrotropin (TSH) (Fig 1D). Open circles represent percents of GDM cases at each decile of the variable shown on the X-axis. Solid lines indicate unadjusted slopes of the respective relationships; p-values indicate slope significance. The dotted line in Fig 1C shows the slope of the fT4/GDM relationship, after adjustment for age, weight, and TSH.

Analyses included descriptive statistics, t-tests for continuous variables, and chi square tests for categorical variables. We used stepwise regression to adjust fT4 for the covariates age, weight, and TSH, after logarithmic transformation. We used linear regression to establish the relationship between GDM and the variables age, weight, and TSH; p-values in the figure are for slope significance (Fig 1). We defined unadjusted and adjusted odds ratios for GDM by comparing rates at the first fT4 decile vs. the remaining deciles. Statistical analyses were performed using SAS 9.4 (Cary, NC, USA).

## Results

Table 1 shows selected characteristics of 9,079 women without GDM compared to 272 women with GDM (TSH 2^nd^ to 98^th^ centile). Women with GDM are older, weigh more, deliver earlier, and a greater percentage smokes cigarettes. Table 2 shows that median TSH and fT4 are lower at 15–18.9 weeks’, but not at 11–14 weeks’ gestation. The rate of TPO antibody positivity does not differ significantly between the two groups.

**Table 1:** Characteristics of women with and without gestational diabetes.

Characteristic*	No GDM	GDM	P
**Participating Women (n)**	9,079	272	N/C
**Gestational age (weeks)**	16.1 (0.9)	16.1 (0.8)	0.37
**Age (years)**	28·0 (5·5)	32.0 (5·6)	<0·0001
**Weight (kg)**	64.5 (14.2)	75·0 (21.3)	<0·0001
**Education (years)**	14·6	14.6	0·67
**Education (% ≤12 years)**	18.1	15.4	0·21
**Primigravid (%)**	41.9	39.7	0.47
**Smokes cigarettes (%)**	3.0	5.1	0.04
**Gestational age at delivery (weeks)**	39.1 (2·2)	38.7 (2.0)	<0·0001
**Gestational age (% <37 weeks)**	7.0	10.3	0·04
**Gestational age (% <34 weeks)**	2.3	2.9	0·52
**Birthweight (grams)**	3,401 (510)	3,430 (608)	0.33
**Birthweight (% <2500 grams)**	4.9	5.9	0.47
**Birth weight (% ≥4000 grams)**	8.7	11.0	0.18

*All characteristics are reported as Median (SD) unless specified otherwise

**Table 2:** Thyrotropin (TSH), free thyroxine (fT4) and thyroperoxidase (TPO) antibodies among women with and without gestational diabetes (GDM).

	No GDM	GDM	P
11–14 weeks’ gestation*			
** TSH (mIU/L)**	1.05 (0.40)	0.99 (0.38)	0.46
** fT4 (pmol/L)**	14.19 (0.13)	13.80 (0.09)	0.30
** TPO antibody >35 IU (%)**	8.6	9.2	0.72
15–18.9 weeks’ gestation*			
** TSH (mIU/L)**	1.23 (0.26)	1.15 (0.30)	0·026
** fT4 (pmol/L)**	13.03 (0.14)	12.38 (0.10)	<0.01
** TPO antibody >35 IU (%)**	8.1	9.2	0.53

*values reported as Median (log SD), unless specified otherwise

Table 3 applies stepwise regression to examine interactions between fT4 (dependent variable) and other characteristics in Tables 1 and 2 that differ significantly between women with and without GDM. Age and weight show the strongest interactions with fT4. TSH shows only weak interaction (r^2^ = 0.0014) but is included on the list of variables to be considered in adjusting the fT4-GDM relationship, due to its known association with fT4. When the lowest fT4 decile is compared with the 2^nd^-10^th^ deciles at 15–18.9 weeks’ gestation, the odds ratio (OR) for GDM is 2.06 [95% CI 1.37–3.09] (unadjusted); and 1.89 [95% CI 1.26–2.84] (adjusted for weight, age, and TSH). Adding TPO antibody to the adjustment model does not alter the relationship [1.87 (1.24–2.81)]. At 11–14 weeks’ gestation, the OR for GDM is 1.45 [95% CI 0.97–2.16] (unadjusted); and 1.11 [0.74–1.65] (adjusted).

**Table 3:** Extent to which variables that differ significantly between women with and without gestational diabetes might influence fT4 concentration (9,079 women without gestational diabetes).

Variable	Partial r^2^
Age*	0.0283
Weight*	0.0136
TSH*	0.0014
**Education**	0.0013
**Smoking**	0.0001

*Data transformed logarithmically prior to analysis

Fig 1 shows that weight (Fig 1A), age (Fig 1B), and fT4 (Fig 1C) are associated univariately with GDM in decreasing order of significance. TSH (Fig 1D) is not significantly associated. The dashed line in Fig 1C indicates that the association between fT4 and GDM remains significant after adjustment for the other three variables.

## Discussion

**Fig 2:**
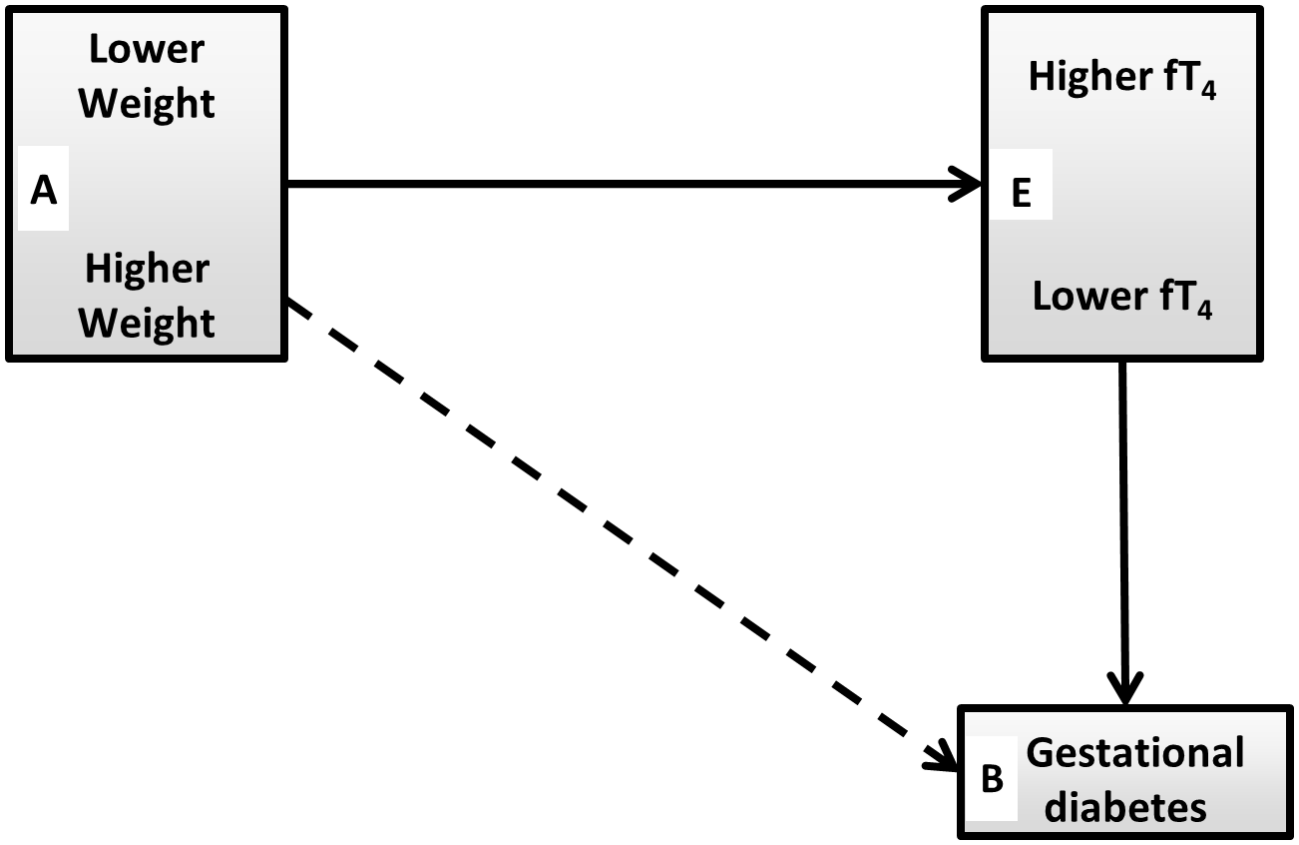
Second trimester relationships between free thyroxine (fT4), maternal weight, and gestational diabetes in the FaSTER trial. A to E Indicates that there is an inverse relationship between maternal weight and fT4 [13, 14, 21]; E to B Indicates that lower fT4 is associated with a higher GDM rate [7]; A to B Indicates that higher weight is associated with higher GDM rate.

An inverse relationship between fT4 and weight during pregnancy has been recognized for several years [18–20], including an association between low fT4 and high weight in the second trimester among euthyroid women in the FaSTER trial [14]. In a separate FaSTER analysis, fT4 was found once again to decrease with increasing weight among hypothyroid women being treated with L-thyroxine to maintain a euthyroid state, suggesting a link between weight and deiodinase activity [21]. In the present FaSTER analysis, high maternal weight and low fT4 are both associated with increased GDM risk (Fig 2).

Studies from several centers between 2000 and 2015 report that low fT4 levels are significantly associated with GDM in the second and third trimesters [6–9], but not in the first [7, 9, 10]. In the FaSTER dataset, a significant relationship is also present between fT4 and GDM during the second trimester (15–18.9 weeks’ gestation), which does not, however, reach significance in the first trimester, suggesting a progression toward glucose intolerance as pregnancy progresses.

Thyroid hormones play an important role in glucose metabolism, but triiodothyronine (T3), rather than T4, is the biologically active hormone primarily responsible for glucose-related metabolic activity [22, 23]. Hyperglycemia typically occurs when thyroid hormone levels are elevated, rather than low. When hyperthyroidism develops in the presence of diabetes, for example, the attendant refractory hyperglycemia accompanying type 2 diabetes is reversed only after a return to the euthyroid state [22]. Given this circumstance, it is not immediately obvious why low fT4 should qualify as a candidate agent for contributing to GDM risk in euthyroid women.

A biologically plausible explanation for this apparent paradox comes from two population-based studies in pregnant women that found not only a reciprocal fT4/BMI relationship, but also a direct relationship between maternal free T3 (fT3) and BMI [18, 19]. Among 5,072 Finnish women, fT4 decreased, and fT3 increased, monotonically as BMI increased, each changing about 5% between a BMI of <20 kg/m^2^ and >30 kg/m^2^ (both P <0.001) [19]. Among 3,592 pregnant women in London, multiple regression analysis showed a reciprocal relationship between Log_10_ fT4 and BMI and a direct relationship between Log_10_ fT3 and BMI (both P <0.0001) [18]. In both studies, therefore, lower fT4 associated with obesity appears to occur in conjunction with higher fT3.

Two other observational studies add further insight, by examining fT4 levels and fT3/fT4 ratios in relation to biometric and metabolic parameters in euthyroid pregnancies [24, 25]. The fT3/fT4 ratios serve as a proxy for peripheral deiodinase activity. Both studies documented not only a reciprocal relationship between fT4 and BMI, but also a direct relationship between the fT3/fT4 ratio and BMI, indicating increased peripheral deiodinase activity.

In the first study, Bassols et al recruited 321 women with singleton pregnancies at 24–28 weeks’ gestation [24]. Women with GDM, preeclampsia, and fetal malformations or asphyxia were excluded. The highest hemoglobin A1c (HbA1c), fasting plasma insulin (FPI), homeostasis model assessment-insulin resistance (HOMA-IR), maternal BMI, placental weight, and triglyceride levels occurred in the lowest fT4 quintile and in the highest fT3/fT4 ratio quintile. With the exception of placental weight, these relationships were stronger when analyzed by fT3/fT4 ratio than by fT4 alone. In the second, Knight et al studied 965 women without either GDM or known thyroid disease and documented significant inverse correlations between fT4 and maternal BMI, HbA1c, FPI, HOMA-IR, and triglycerides, at 28 weeks’ gestation [25]. Once again, direct correlations of stronger significance were found between the fT3/fT4 ratio and these same metabolic parameters.

Beginning in the 1970s, it was recognized that most circulating T3 was generated via peripheral conversion of T4 and that a direct correlation existed between T3 and body weight in non-pregnant adults [26]. All T4 and 20% of T3 is produced in the thyroid gland; the remaining 80% of T3 is converted peripherally by deiodinase activity via mono-deiodination of T4 [27]. Peripheral conversion of T4 to T3 decreases dramatically in the presence of extreme caloric deprivation [28–31] and increases with overfeeding [32], indicating that peripheral deiodinase activity is highly responsive to acute changes in caloric intake. In 2014, Agnihothri et al carried out a 12 month reduced calorie dietary intervention study in non-pregnant adults. T3 decreased significantly with longer term weight loss, and the T3/fT4 ratio decreased significantly with >5% loss of body weight, leading to the conclusion that decreased peripheral conversion of T4 to T3 at least partially explained the longer term dietary and weight changes in thyroid hormone homeostasis [33].

In the above human studies, peripheral deiodinase activity could only be assessed via surrogate measures in serum or plasma (*e*.*g*., T3/T4 ratios, reverse T3), due to the impracticality of obtaining tissue samples. Direct measurements of hepatic deiodinase-1 mRNA and deiodinase-1 activity in animal studies, however, support conclusions drawn from surrogate measurements. A high fat diet in wild type mice, for example, induces not only lower serum T4 and higher T3 and T3/T4 ratios, but also higher deiodinase-1 mRNA and activity in the liver [34]. Although the FaSTER trial did not include a dietary assessment, higher weight is a reasonable indicator of over-nutrition. Women were early in the second trimester at the time of sampling, and most were likely following their usual dietary routines and caloric intake. The reciprocal relationship between fT4 and maternal weight observed in FaSTER and other studies [18, 19, 24, 25], therefore, should reflect a generally steady dietary state within the pregnancy population. A recent FaSTER analysis documented a similar reciprocal fT4/weight relationship among 306 pregnant women being treated for hypothyroidism [21].

An important limitation of the present study is that fT3 was not measured as part of the original study’s protocol, and samples are no longer available to carry out retrospective analyses. As a consequence, it has been necessary to rely on results from other studies to fully interpret our findings. Strengths include a large cohort of pregnancies, with fT4 and TSH measured on all of the women and with complete ascertainment of GDM cases, along with relevant demographic data. Reference data from the FaSTER dataset have been published for fT4 and TSH, verifying reliable performance [7, 13, 14, 17]. Documentation of assay performance and availability of a large study population are both important, because direct immunoassays for fT4 are occasionally affected by altered concentrations of binding proteins, such as albumin and thyroxine binding globulin.

**Fig 3:**
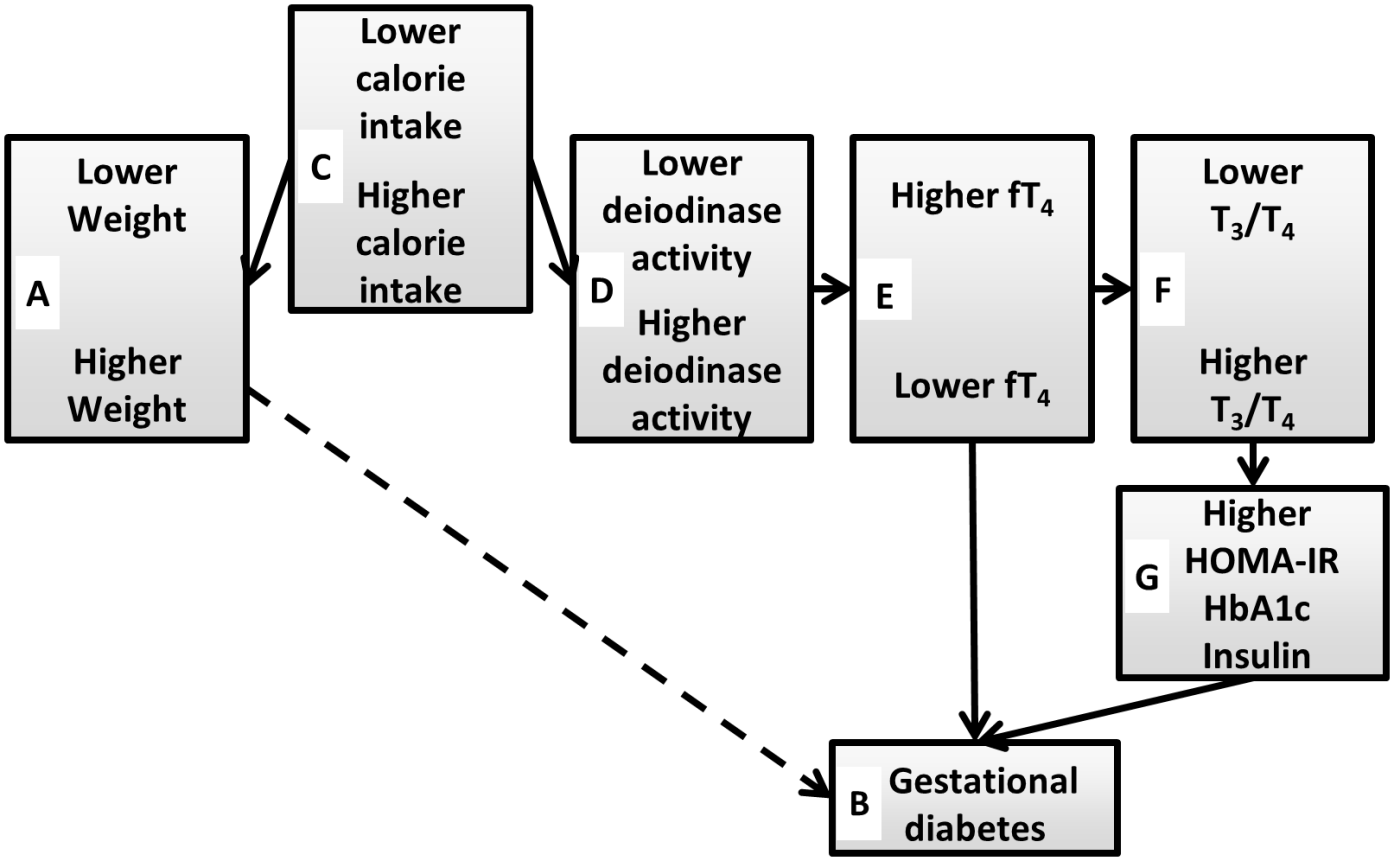
Schematic diagram depicting how caloric intake and deiodinase activity fit into relationships shown in Fig 2. Higher caloric intake (C) reflects higher weight (A) and induces higher deiodinase activity (D) [32, 34]. Lower fT4 (E) and higher T3/T4 ratios (F) occur as a consequence of higher deiodinase activity and are associated with both insulin resistance (G) [24, 25] and gestational diabetes (B).

In this study, we report a new finding (Fig 3) that links obesity with induction of triiodothyronine (T3), a candidate causal agent contributing to gestational diabetes among women with normal thyroid gland function (euthyroid); T3 is the hormonal product responsible for stimulating endogenous glucose production. In the present circumstance, normal amounts of thyroxine (T4), the inactive thyroid hormone precursor, are produced by the gland. Peripheral deiodinase activity (*e*.*g*., liver, muscle) then uses T4 as its substrate to produce the active hormone (T3). Obesity induces increased deiodinase activity, as indicated by a combination of higher T3 and lower T4 levels. Our study uses free T4 levels as the marker for deiodinase activity; T3 was not available in our study (please see limitations, above). At present, ectopic accumulation of fat in liver and muscle associated with over-nutrition is the best understood causal agent for insulin resistance and type 2 diabetes (via diacylglycerol-induced inhibition of insulin receptor substrate) [35]. In our study, increased caloric intake is also unifying feature, in this case connecting higher weight on the one hand with deiodinase activity and gestational diabetes on the other. It thus appears that over-nutrition might predispose to both insulin resistance (ectopic fat) and endogenous glucose production (increased deiodinase), adding another dimension to the complexities of gestational diabetes.

## Supporting Information

S1 Dataset Dataset forming the basis for analyses in the FaSTER trial for the present study. (XLS)
